# MicroRNAs differentially expressed in Behçet disease are involved in interleukin-6 production

**DOI:** 10.1186/s12950-016-0130-7

**Published:** 2016-07-19

**Authors:** Min-Yeong Woo, Su Jin Yun, Okki Cho, Kyongmin Kim, Eun-So Lee, Sun Park

**Affiliations:** Department of Microbiology, Ajou University School of Medicine, Youngtongku Wonchondong San 5, Suwon, 442-749 Korea; Department of Biomedical Sciences, The Graduate School, Ajou University, Youngtongku Wonchondong San 5, Suwon, 442-749 Korea; Department of Dermatology, Ajou University School of Medicine, Youngtongku Wonchondong San 5, Suwon, 442-749 Korea

**Keywords:** Behçet syndrome, Cytokine, Gene expression, Pathogenesis, microRNA

## Abstract

**Background:**

Behcet’s disease (BD) is characterized by systemic recurrent inflammation with increased production of tumor necrosis factor (TNF)–α and interleukin (IL)-6 by peripheral blood mononuclear cells (PBMCs). To gain insight into the underlying mechanisms of this disease, the expression levels of distinct microRNAs in PBMCs of BD patients were determined and their association with TNF-α and IL-6 production was evaluated.

**Findings:**

The expression levels of microRNAs, miR-638 and miR-4488, were reduced in patients with stable BD in comparison with healthy controls. In addition, the expression of miR-3591-3p was increased in patients with active BD when compared to patients with stable BD. Transfection of miR-638 and miR-4488 inhibitors, together with miR-3591-3p mimics, increased IL-6 mRNA levels in THP-1 cells in response to LPS stimulation.

**Conclusions:**

We observed differential expression of microRNAs associated with increased production of IL-6 in BD patients.

**Electronic supplementary material:**

The online version of this article (doi:10.1186/s12950-016-0130-7) contains supplementary material, which is available to authorized users.

## Background

Behçet disease (BD) is a relapsing inflammatory disease characterized by oral ulcers, genital ulcers, skin lesions and ocular inflammation [1]. It has been proposed that the development of BD is associated with barrier dysfunction and abnormal innate immune reactions in environmentally exposed organs, triggering neutrophilic inflammation [2]. Expression of proinflammatory cytokines in peripheral blood monocytes is increased compared to healthy controls at basal state and in response to stimulation with lipopolysaccharide (LPS) [3]. Currently, TNF-α neutralizing antibodies are used to treat BD. Based upon the experience of several case reports, IL-6 neutralizing antibodies seem also to be promising for BD patients with refractory uveitis and central nervous system involvement [4]. However, the underlying mechanisms for increase in proinflammatory cytokine levels remain elusive.

MicroRNAs, regulating gene expression through translational inhibition of target genes, are involved in autoimmune diseases. Mice that are deficient in miR-155 are highly resistant to experimental autoimmune encephalitis [5]. A role for microRNAs has been suggested in several autoimmune diseases, including BD [6].

In this study, we analyzed the expression of selected microRNAs in PBMCs of BD patients and investigated their roles in the expression of TNF-α and IL-6.

## Methods

### Study subjects

Patients with BD who visited the Department of Dermatology at the Ajou University Hospital and were diagnosed according to the International Study Group and Japanese criteria for BD were enrolled in this study. The patients with active BD exhibited at least one of the symptoms of BD, despite the medication, whereas the symptoms were well-controlled in the patients with stable BD. Clinical features and medications at the time of inclusion are summarized in Table 1. Healthy volunteers were included as a control group. All subjects provided informed consent for the study, which was approved by the local Institutional Review Board (AJIRB-GEN-GEN-10-119).

**Table 1:** Characteristics of healthy controls and patients with BD

Demographics	Controls (*n* = 8)	Stable BD (*n* = 10)	Active BD (*n* = 11)
Age (mean age ± SD)	29.3 ± 4.5	43.2 ± 11.0	44.8 ± 4.3
Sex (male : female)	4:4	7:3	5:6
Clinical features of BD patients		Case No (%)	
Oral ulcer			1 (9.1)
Erythema nodosum			6 (54.5)
Ocular symptoms			4 (36.4)
Arthralgia			1 (9.1)
Gastrointestinal involvement			1 (9.1)
Neurological involvement			1 (9.1)
HLA-B51		5 (50)	6 (54.5)
Medication			
Immunosuppressive agents^a^		2 (20)	7 (63.7)
Colchicine		10 (100)	10 (90.9)
other antiinflammatory agents^b^		9 (90)	10 (90.9)
Minocycline		1 (10)	5 (45.5)

^a^ prednisolone or azathioprine

^b^ salazopyrin or pentoxyfylline or aspirin

### Cell stimulation

PBMCs were separated using Ficoll-Paque Plus (StemCell Technologies, Vancouver, Canada) and CD11b + cells were isolated using magnetic beads (Miltenyi Biotec, Bergisch Gladbach, Germany). The purity of CD11b + cells was analyzed by flow cytometry. The cells (3 × 10^6^/mL) were stimulated with 10 ng/mL LPS (Sigma-Aldrich, St Louis, MO) for 3 h in RPMI1640 medium (Invitrogen, Gaithersburg, MD) containing 10 % fetal bovine serum (Invitrogen), penicillin (100 U/mL, Invitrogen) and streptomycin (100 μg/mL, Invitrogen).

### Real-time reverse transcription-polymerase chain reaction (qRT-PCR)

Total RNA was extracted with TRIzol (Invitrogen). qRT-PCR was performed using specific primers for IL-6 and TNF-α. For quantification of microRNA, standard TaqMan small RNA assay kit and the TaqMan Universal PCR Master Mix (Applied Biosystems) were used. RNU-66 was used to normalize the PCR data. PCR was run on an ABI PRISM 7000 Sequence Detection System.

### Transfection of microRNA mimics and inhibitors

THP-1 cells (2.5 × 10^4^) were transfected with a 40 nM mixture of equal amount of miR-638 inhibitor, miR-3591-3p mimic and mir-4488 inhibitor (Ambion, Carlsbad, CA, USA) or a mixture of control mimics and inhibitors using lipofectamine 2000 (Invitrogen). FAM-conjugated control mimics (1/10 of the total amount of mimics and inhibitors) were cotransfected to determine the transfection efficiency. Cells were stimulated with LPS (10 ng/ml) for 3 h at 21 h post transfection and then subjected to RNA isolation.

### Statistics

Kruskal-Wallis test with Dunn’s procedure for multiple comparison or Student’s *t*-test were used to determine *P* values. Differences with a *P*-value < 0.05 were considered significant.

## Results and discussion

**Fig. 1:**
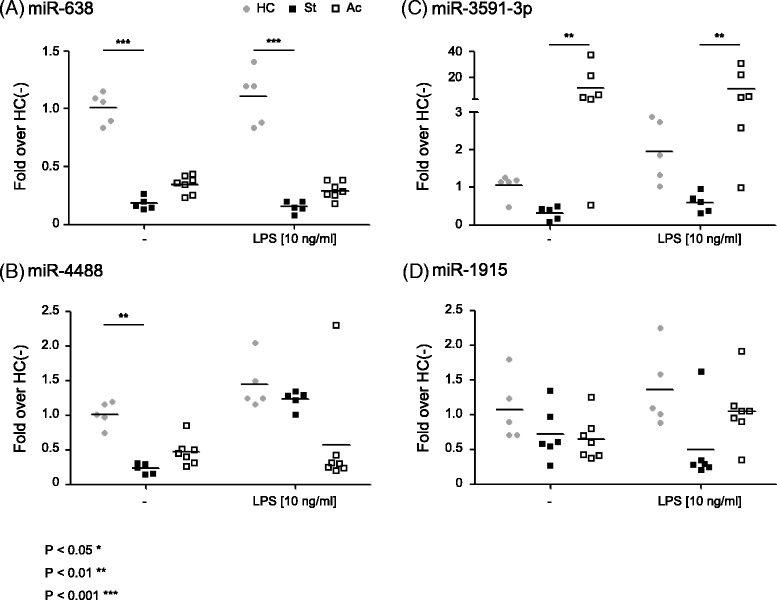
Differential expression of microRNAs in PBMCs from patients with Behcet’s disease (BD). PBMCs from healthy controls (HC, *n* = 5), stable BD patients (St, *n* = 5 or 6) and active BD patients (Ac, *n* = 6 or 7) were stimulated with LPS (10 ng/mL) for 3 h or left untreated. Expression levels of miR-638 (**a**), miR-4488 (**b**), miR-3591-3p (**c**) and miR-1915 (**d**) were analyzed by qRT-PCR. Fold over HC(−): Relative microRNA levels versus the average microRNA level in HC PBMCs without LPS stimulation. Each symbol represents a single subject. Bars represent the mean of each group. **P < 0.01, ***P < 0.001. *P* values were calculated using the Kruskal-Wallis test with Dunn’s procedure for multiple comparisons

Preliminary results from a microRNA microarray analysis using RNA samples isolated from PBMCs of BD patients suggested that six microRNAs were differentially expressed between BD patients according to the levels of IL-6 and TNF-α expression (data not shown). We therefore examined the expression levels of four selected microRNAs (miR-638, miR-4488, miR-3591-3p and miR-1915) using real-time RT-PCR (Fig. 1). Compared to healthy controls, miR-638 expression was 5-fold reduced in PBMCs from stable BD patients regardless of LPS stimulation (*p* < 0.001). Expression of miR-4488 was approximately 25 % that of healthy controls in PBMCs from stable BD patients, in the absence of LPS stimulation (*p* < 0.01). LPS stimulation increased miR-4488 levels in PBMCs from stable BD patients to the levels observed in PBMCs from healthy controls. The levels of miR-3591-3p in PBMCs from stable BD patients were approximately one third of those of healthy controls both in the presence and absence of LPS stimulation, even though there was no statistical significance. In contrast, miR-3591-3p expression in PBMCs of active BD patients was significantly increased compared to stable BD patients (*p* < 0.01). Expression levels of miR-1915 were not significantly different between study groups. The expression of these microRNAs was not associated with the clinical symptoms, HLA-B51 genotype or medication.

**Fig. 2:**
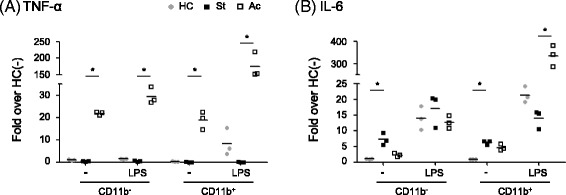
Differential mRNA expression of proinflammatory cytokines in CD11b + and CD11b- cells from BD patients. CD11b + and CD11b- cells isolated from 3 healthy controls (HC), 3 stable BD patients (St) and 3 active BD patients (Ac) were cultured with or without LPS for 3 h. mRNA levels of the indicated genes were analyzed by qRT-PCR. Fold over HC(−): Relative mRNA level versus the average mRNA level in CD11b- cells of HC without LPS stimulation. Each symbol represents a single subject. Bars represent the mean of each group. *P < 0.05. *P* values were calculated using the Kruskal-Wallis test with Dunn’s procedure for multiple comparisons

We then determined the levels of TNF-α and IL-6 mRNA in CD11b^+^ and CD11^−^ cell populations from PBMCs of BD patients (Fig. 2). TNF-α expression was significantly increased in both cell populations from patients with active BD when compared to patients with stable BD, regardless of LPS stimulation (*p* < 0.05). In the absence of LPS stimulation, IL-6 expression was significantly increased in both cell populations from patients with stable BD compared to healthy controls. However, LPS-stimulated IL-6 expression was significantly upregulated in CD11b + cells from patients with active BD compared to those with stable BD (*p* < 0.05).

**Fig. 3:**
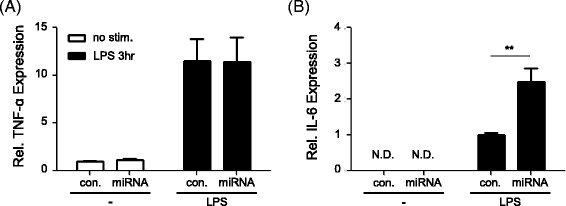
Upregulation of IL-6 transcript levels by transfection of microRNA inhibitors and mimics. THP-1 cells (2.5 × 10^4^) were transfected with inhibitors for miR-638 and miR-4488 together with mimics for miR-3591-3p (miRNA) or with a mixture of control inhibitors and mimics (con). PAM-control microRNA (1/10) was cotransfected to determine the transfection efficiency. After 21 h, cells were stimulated with LPS (10 ng/mL) for 3 h and mRNA levels of TNF-α (**a**) and IL-6 (**b**) were assessed by qRT-PCR. Independent experiments were repeated more than three times in duplicate or quadruplicate. Relative TNF-α expression: mRNA level/average mRNA level of con in the absence of LPS. Relative IL-6 expression: mRNA level/average mRNA level of con in the presence of LPS. N.D.; not detected, **P < 0.005. Student’s *t*-test was used for the calculation of *P* values

To understand the relevance of these microRNAs in inflammatory cytokine production, we transfected THP-1 cells with a mixture of inhibitors for miR-638 and miR-4488 together with mimics of miR-3591-3p, and then analyzed mRNA levels of TNF-α and IL-6 (Fig. 3). Compared to cells transfected with a mixture of control microRNA mimics and inhibitors, LPS-stimulated IL-6 mRNA levels were approximately 2.5 fold increased in cells transfected with inhibitors of miR-638 and miR-4488 together with mimics of miR-3591-3p (*p* < 0.05); however, TNF-α mRNA levels were not different. Additionally, neither a combination of inhibitors of miR-638 and miR-4488, nor a combination of mimics of miR-3591-3p, with either a miR-638 inhibitor or a miR-4488 inhibitor, affected IL-6 mRNA levels in transfected cells (data not shown). Taken together, differential expression of miR-638, miR-4488 and miR-3591-3p was observed in PBMCs of BD patients and its association with IL-6 expression was demonstrated.

Recently, differential expression of microRNAs in BD has been reported. miR-155 expression has been found to be decreased in BD with uveitis compared to that in the healthy controls [7]. Additionally, miR-23b was decreased in CD4+ T cells of BD patients with active uveitis. This decrease was suggested to be involved in Th17 response through the activation of the Notch pathway [8]. Differential expression of miR-720 and miR-139-3p in PBMCs from BD compared to healthy controls has been published [9]. Our study is the first to discover altered expression of miR-638, miR-4488 and miR-3591-3p associated with BD. Differential expression of these microRNAs has been associated with several disorders, such as breast carcinoma and systemic lupus erythematosus for miR-638 [10, 11], and Barrett’s esophagus for miR-4488 [12]. However, little information is currently available on the role of these microRNAs. Identified targets for miR-638 include BRCA1, sex determining region Y (SRY)-box (SOX) 2, cyclin-dependent kinase 2 and tumor protein p53 inducible nuclear protein 2, human (TP53INP2), which are involved in proliferation, apoptosis and DNA repair in tumor cells [10, 13–15]. Given that infections with viruses such as hepatitis B virus, hepatitis C virus and Chikungunya virus increase miR-638 expression [16] and that herpes virus is believed to be involved in BD pathogenesis, it is possible that underlying viral infection and/or associated inflammation affect microRNA expression in patients with BD. Although further studies are required to understand the implications of differential expression of these microRNAs in BD pathogenesis, we demonstrated that inhibitors of miR-638 and miR-4488 together with miR-3591-3p mimics could upregulate IL-6 mRNA levels.

In conclusion, our results demonstrated differential expression of microRNAs in PBMCs from patients with BD and suggested that these molecules played a regulatory role in the production of IL-6. Further studies regarding the mechanisms underlying differential expression of these microRNAs in PBMCs from patients with BD is warranted to elucidate the pathogenesis and identify new therapeutic targets for BD.

## Abbreviations

BD, Behçet’s disease; HC, healthy controls; IL-6, interleukin-6; LPS, lipopolysaccharide; PBMCs, peripheral blood mononuclear cells; qRT-PCR, real-time reverse transcription-polymerase chain reaction; SD, standard deviation; TNF - α, tumor necrosis factor – α

## Additional file

Additional file 1: Table S1. Differentially expressed microRNAs between BD responders and nonresponders to colchicine treatment. (DOCX 11 kb)
