# Supplementary material for: MicroRNAs differentially expressed in Behçet disease are involved in interleukin-6 production
Source: J Inflamm (Lond). 2016 Jul 19;13:22. doi: 10.1186/s12950-016-0130-7 (PMC4952146; doi:10.1186/s12950-016-0130-7)
Supplement: Additional file 1: Table S1. — Differentially expressed microRNAs between BD responders and nonresponders to colchicine treatment. (DOCX 11 kb) [file 12950_2016_130_MOESM1_ESM.docx]

**Additional file 1: Table S1** Differentially expressed microRNAs between BD responders and nonresponders to colchicine treatment

| **Stimulation** | **miRNA** | ***P*-value** | **log2 (R/NR^a^)** |
| --- | --- | --- | --- |
| None | miR-638 | 0.037 | 1.30 |
| LPS | miR-4668-5p | 0.020 | -7.66 |
|  | miR-4466 | 0.022 | 2.06 |
|  | miR-3591-3p | 0.035 | -3.14 |
|  | miR-4488 | 0.035 | 2.08 |
|  | miR-1915 | 0.040 | 2.42 |

Altered cytokine expression levels have been reported in unstimulated and LPS-stimulated BD PBMCs compared to healthy controls (Br J Dermatol 167 (2012) 914-921). MicroRNA profiles of BD PBMCs stimulated with LPS (10 ng/mL) for 3 h or not were analyzed by microarray.

^a^R, responder to colchicine treatment; NR, nonresponders to colchicine treatment.
